# Alzheimer's diagnosis beyond cerebrospinal fluid: Probe-Free Detection of Tau Proteins using MXene based redox systems and molecularly imprinted polymers

**DOI:** 10.1016/j.biosx.2024.100513

**Published:** 2024-10

**Authors:** Ajith Mohan Arjun, Sudhaunsh Deshpande, Tom Dunlop, Beth Norman, Daniela Oliviera, Georgeta Vulpe, Felismina Moreira, Sanjiv Sharma

**Affiliations:** a Dept. of Biomedical Engineering, Faculty of Science and Engineering, Swansea University, UK; b The Advanced Imaging of Materials (AIM) Facility, Faculty of Science and Engineering, Swansea University, UK; c CIETI - LabRISE-School of Engineering, Polytechnic of Porto, R. Dr. António Bernardino de Almeida, 431, 4249-015, Porto, Portugal

## Abstract

Phosphorylated Tau proteins are promising biomarkers for the diagnosis and prognosis of Alzheimer's disease. This study presents a novel voltametric sensor using a vanadium MXene polydopamine (V_x_PDA) redox active composite and a Tau-441-specific polyaniline molecularly imprinted polymer (PANI MIP) for the sensitive detection of Tau-441 in interstitial fluid (ISF) and plasma. The V_x_PDA/PANI MIP sensor demonstrates a broad detection range of 5 fg/mL to 5 ng/mL (122 aM/L to 122 pM/L) in ISF without the use of redox mediators, with a lower limit of detection (LOD) of 2.3 fg/mL (60 aM/L). Furthermore, a handheld device utilizing this technology successfully detects Tau-441 in artificial serum with high sensitivity (5 fg/mL to 150 fg/mL (122 aM/L to 366 aM/L)) and specificity within a clinically relevant range. The rapid detection time (∼32 min) and low cost (∼£20/device) of this sensor highlight its potential for minimally invasive, early AD diagnosis in clinical settings. This advancement aims to facilitate a transition away from invasive cerebrospinal fluid (CSF)-based diagnostic techniques for AD.

## Highlights

• Novel Vanadium MXene-Based Sensor enables probe-free biomarker detection.
• Performance validated in interstitial fluid and artificial serum.
• Ultrasensitive Detection of 122 aM/L of Tau-441.
• Rapid (∼32 min) Tau-441 detection in serum for ∼£20.
• Simplified, accessible early Alzheimer's diagnostics.

## 1 Introduction

Alzheimer's disease (AD) is a progressive neurodegenerative condition, which affects the brain. It is one of the most common causes of dementia in the world. It was reported by Gustavsson et al. that approximately 416 million people suffered from various stages of this disease and this number was estimated to be about 20% of all people aged 50 and above (Gustavsson et al., 2023). Additionally, it was reported by the Alzheimer's association that health care and long terms costs of people suffering from AD were estimated to be at $360 million in 2024 with projections equalling $ 1 trillion by 2050. Most common biomarkers associated with AD include the presence of intracellular phosphorylated tau tangles (p-tau) and extracellular amyloid beta plaques (Aβ) (Muir et al., 2024). The Aβ plaques accumulate initially in the cerebral regions and spread to the neocortex and eventually reach the cerebellum. While plaque accumulation serves as a trigger of downstream pathways, tau pathologies colocalize with cortical atrophy and can be used as a predictor of future neurodegeneration (Hampel et al., 2021; Vogel et al., 2021).

As part of the first steps towards initiating treatment protocols at the earliest stages for AD, it is essential to utilize highly sensitive diagnostic protocols (Dubois et al., 2021). Currently, positron emission tomography (PET) scans and detection of biomarkers from cerebrospinal fluid (CSF) are the gold standard methods for the diagnosis of AD (Kas et al., 2020). The PET scans utilize a radioactive tracer drug, which is injected into the body of the subject. These radioactive tracer drugs cause health effects like nausea, vomiting, hypersensitivity, and in adverse cases kidney failure (Yang et al., 2024). Additionally, the detection of AD biomarkers from CSF can be achieved only through extraction of CSF through the lumbar puncture methods, which represents a painful extraction technique (Liu et al., 2022). Additionally, these techniques require long durations to obtain accurate diagnosis, in addition to being resource intensive both in terms of cost and man power requirement (Wittenberg et al., 2019). The ectodermal origin theory, postulates an antenatal relation between the brain and the skin in addition to proving shared molecular factors between both the skin and the brain (Jameson et al., 2023). Also, the two-compartment model postulates the presence of blood-based biomarkers in dermal interstitial fluid (ISF) (Friedel et al., 2023). Due to a combination of these models, it can be confirmed that the biomarkers present in the CSF are also present in the blood and the skin compartment. These theories present a viable alternative to alleviate the demerits associated with PET scans and CSF extraction. Minimally invasive techniques like blood based and ISF based detection of AD biomarkers present a promising strategy in this scenario. There have been extensive reports that use blood and ISF for detection of biomarkers in these matrices (Friedel et al., 2023; Yao et al., 2021). These include electrochemical (Rawson et al., 2019; Vulpe et al., 2024), optical (Qu et al., 2024), electrical and electronic (Ok et al., 2024; Wang et al.), and impedance based techniques (Banga et al., 2023). Among these techniques, electrochemical methods offer benefits in terms of superior sensitivity, ease of electrode fabrication, and potential for commercialisation. Over the last 10 years, the analysis of AD biomarkers from blood and ISF has shown great potential due to its simplicity, diagnostic accuracy (95%–97%), and cost effectivity. Low concentrations of these biomarkers have been detected using composites of nanomaterials and bio-receptors (Fahmy et al., 2022).

The use of nanomaterials for achieving highly sensitive detection of biomarkers in complex matrices has been reported extensively (Wu et al., 2023). Specifically, two dimensional (2D) materials have been reported for these applications. The use of 2D materials has been reported to increase loading of redox active nanoparticles and bioreceptors thereby leading to superior sensitivity (Bolotsky et al., 2019). The advent of transition metal carbides and nitrides (MXenes), which possess properties superior to those 2D materials like graphene in terms of solution processibilities and conductivities has led to the use of MXenes for a plethora of applications (Naguib et al., 2012). The use of these MXenes has resulted in better sensitivity, ease of fabrication, and cost effectiveness (Arjun et al., 2023; Rasheed et al., 2022). However, restacking of MXenes sheets is a challenge, which has attracted significant attention. To alleviate this demerit methods like formation of composites of MXenes has been proposed. These composites, help in preventing the restacking of MXenes, improving conductivities, and achieving superior electron transfer rates (Mohan Arjun et al., 2023; Zhao et al., 2019). The use of MXenes like vanadium MXenes has also proven to be biocompatible as reported in recent studies by Fusco et al. (2023). The use of biocompatible MXenes for sensing applications in blood and ISF would help in achieving superior sensing performance, cost effective fabrication, and also better commercial viability. Additionally, MXenes have been reported to serve as efficient immobilisation platforms for a wide of biomolecules for electrochemical sensing applications (Ramanavicius and Ramanavicius, 2020). In this manuscript, we have reported on the synthesis of a vanadium MXene polydopamine (V_x_PDA) composite, which not only possess the superior electrochemical properties of MXenes but is also biocompatible. The presence of PDA film also prevents the restacking of these MXenes, which is another merit of using this material.

Bioreceptors play a pivotal role in Alzheimer's disease (AD) diagnosis, serving as molecular tools that detect specific biomarkers associated with the disease. Bioreceptors are typically biological molecules, such as antibodies or aptamers, engineered to bind specifically to these biomarkers with high affinity and selectivity. Antibodies present a class of bioreceptors that have been explored extensively for the detection of AD biomarkers. By harnessing the binding interactions between antibodies and target biomarkers, diagnostic assays can be developed to detect and quantify the presence of AD-related molecules in patient samples. However, the use of these antibodies requires the use of complex immobilisation procedures and sensing strategies like label-based techniques for sensing of the binding event between the antibody and the analytes, which makes sensor operation complex (Scarano et al., 2016). In such a scenario, the use of molecularly imprinted polymers (MIPs) represents an innovative approach in biosensing and diagnostic technology, offering distinct advantages compared to antibodies. These MIPs are synthetic polymers designed to recognize and bind to target molecules with high specificity. These are synthesized through a process called molecular imprinting, where template molecules are incorporated into a polymer matrix and subsequently removed, leaving behind cavities with shapes complementary to the template molecule. This allows MIPs to selectively bind to target molecules based on their molecular shape and functional groups, offering robustness, stability, and resistance to degradation compared to biological receptors (Li et al., 2024). Additionally, MIPs can be easily synthesized in large quantities, making them cost-effective and scalable for diagnostic applications. MIPs composed of conducting polymers like polyaniline (PANI), polypyrrole (PPy) are amongst the most investigated MIPs for sensing applications (Ayankojo et al., 2024; Ramanavicius and Ramanavicius, 2022). These MIPs have been used extensively for applications involving recombinant severe acute respiratory syndrome coronavirus 2 (SARS-CoV-2) nucleocapsid protein (rN) as reported by Drobysh et al. (2024) and ARS-CoV-2 spike glycoprotein (SARS-CoV-2-S) as reported by Ratautaite et al. (2023). Additionally, there have also been reports involving the use of these materials for diagnosis of neurodegenerative diseases (Pilvenyte et al., 2023).

Among MIPS PANI boasts characteristics such as environmental stability, tuneable morphology, reversible redox chemistry and its electrical conductivity can be controlled (Luo et al., 2017). Furthermore, PANI can be synthesized at low-cost, which is a very attractive characteristic for this research. Due to its superior electrochemical properties, PANI is very efficient in sensing and detecting conductance changes in response to various concentrations of target proteins, and as the backbone of PANI is amine enriched, it offers a plethora of possibilities for immobilizing and binding biomolecules (Riaz et al., 2022). Therefore, in this work, the electropolymerisation was carried out with the functional monomer aniline, which is polymerized to polyaniline to produce a thin, insulating polyaniline film on the MIP based sensor. Most recent reports utilizing MIPs for detection of AD biomarkers have utilized an external redox probe like ferricyanide for electrochemical signal generation. Additionally, these reports have also utilized methods like EIS for the detection of the binding event of these proteins with their corresponding MIPs (Correia et al., 2023; Li et al., 2024; Ren et al., 2023). Although these methods are extensively reported, they present challenges towards commercialisation of devices for blood and ISF based diagnostics (Oliveira et al., 2022). Based on existing reports, we postulate that a voltametric detection of AD biomarkers in blood and ISF would represent commercially viable platforms for achieving blood and ISF based AD detection. Additionally, the dependence on probes like ferricyanide could be overcome by the use of redox active polymers as in built redox active layers in combination with MIPs. There have been multiple reports in this direction like those by Hassine et al. who used Prussian Blue cubes as the redox active layer (Ben Hassine et al., 2023), Lach et al. who used MIPs embedded with ferrocene (Lach et al., 2021), poly (O-phenylenediamine) based MIPs reported by Pereira et al., and ferrocene labelled nanoMIPs reported by Piletsky and coworkers (Alanazi et al., 2021; Lach et al., 2023). Utilizing these considerations, we reported the use of polydopamine as a redox active layer for the detection of Tau-441.

This manuscript reports on the utilization of a PANI based MIP for the detection of Tau-441 in complex matrices like ISF and serum. As a step towards commercialisation, we also present, the implementation of a V_x_PDA composite as a redox active layer for the detection of Tau-441 in these matrices. The V_x_PDA composite was prepared using a facile synthesis strategy and morphological and spectroscopic characterisation revealed the successful modification of the vanadium MXene with PDA. Additionally, the preparation of the V_x_PDA composite was proven to increase the surface area of the electrode, which led to ultrasensitive detection of Tau-441 in both ISF and serum. The V_x_PDA/PANI MIP was able to detect Tau-441 in the range of 5 fg/mL to 5 ng/mL (122 aM/L-122 pM/L) with an LOD of 2.3 fg/mL (∼60 aM/L), which is comparable to existing reports. This sensor surface was also used for the detection of Tau-441 in serum using a handheld device in the range of 5 fg/mL to 150 fg/mL, thus showing its superior sensing performance in low concentrations of Tau-441 in addition to possessing low cost (∼£ 20/device). The handheld device carried out this detection within a short time of ∼32 min using just a drop of the analyte on the electrode surface, resembling a minimally invasive technique for the detection of the Tau-441.

## 2 Experimental

### 2.1 Materials

All chemicals used throughout this project were of analytical grade. Vanadium carbide MXenes were procured from Nanoplexus Limited, UK. Dopamine hydrochloride (H8502), Trizma® hydrochloride (T3253), potassium hexacyanoferrate (II) (K_3_ [Fe(CN)_6_]) (P9387), potassium hexacyanoferrate (III) trihydrate (K_4_ [Fe(CN)_6_]) (244023), DL-Dithiothreitol solution (DTT; 43816), glycerol (G9012), calcium chloride (C1016), potassium chloride (P9541), sodium chloride (S9888), HEPES (H3375), D-glucose (G7021), sodium phosphate monobasic dihydrate (71500), sucrose (S0389), magnesium sulphate (M7506) and phosphate buffered saline (PBS; P4417), were purchased from Sigma. Aniline (158190010), oxalic acid (044410.18, 500 mM), which were used for MIP synthesis and template elution, and 98% H_2_SO_4_ were purchased from Thermo Scientific.

Tau-441 protein (M_w_ 41 kDa) was purchased from AbCAM and was prepared by adding 0.004% DTT and 25% glycerol in PBS buffer to the Tau-441 and stored at −20 °C (Ben Hassine et al., 2021).

Screen printed gold electrodes (SPGE) (220AT and C220AT) were purchased from DropSens.

### 2.2 Preparation of solutions

A 10 mM PBS solution was prepared by dissolving 1 tablet in 200 mL of water. Artificial ISF is prepared by mixing 2.5 mM of calcium chloride, 5.5 mM glucose, 3.5 mM potassium chloride, 10 mM HEPES, 7.2 mM sucrose, 123 mM sodium chloride, 1.5 mM sodium dihydrogen phosphate dihydrate, and 0.7 mM magnesium sulphate. The pH of the solution was then adjusted to pH 7.4 (Ben Hassine et al., 2023). Artificial serum was prepared by mixing 8.035 g of NaCl, 0.355 g of NaHCO_3_, 0.225 g of KCl, 0.231 g of Na_2_HPO_4_, 0.311 g of MgCl_2_, 0.292 g of CaCl_2_, 0.072 g Na_2_SO_4_, 6.118 g of Tris-HCl to 1 L of water (Srinivasan and Rajendran, 2015). Deionised water (resistivity = 18.2 MΩ/cm) was obtained using a Milli-Q water purification.

### 2.3 Material synthesis

Vanadium MXene polydopamine composite (V_x_PDA) was prepared using a protocol modified from previous reports (Chen et al., 2021; Pandey et al., 2024; Yan et al., 2022). Briefly, 2 mg of dopamine hydrochloride was dissolved in Tris-HCl solution (pH 8.5). This was followed by the addition of 20 mg of vanadium MXene. The reaction mixture was kept for 1 h followed by centrifugation for removal of the unreacted constituents.

To design the biosensor, four main steps were carried out. These included the pre-treatment steps, electropolymerisation/imprinting step, template removal and rebinding of the protein to the template cavities created. The pretreatment of the SPGEs were carried out by rinsing with de-ionised water and ethanol following by drying with a stream of air. This was followed by electrochemically cleaning using cyclic voltammetry (CV) from −0.6 V to 0.6 V at a scan rate of 0.1 V/s for 20 cycles in 0.5 M H_2_SO_4_. Imprinting was carried out using CV, after applying 70 μL of the MIP solution directly on to G-SPEs. The MIP solution was made of by mixing 0.2 M aniline and 0.02 mg/mL of the Tau-441 protein. The CV was carried out thrice from −0.2 V to +0.8 V with a scan time of 0.025 V/s. These CVs were observed to confirm the molecular imprinting process (not shown in manuscript). A decrease of peaks in the deposition CVs confirmed the deposition of PANI on the electrode surface. The NIP was fabricated by using the same procedure as the MIP, however the Tau-441 was not included during the fabrication of the NIP.

To remove the template protein, the modified sensors were incubated overnight at −4 °C in the fridge with the addition of 70 μL of 50 mM oxalic acid to the working electrode (Ben Hassine et al., 2023). After incubation, the oxalic acid was removed by washing followed by drying in a stream of air. After removal of the template, the MIPs were stabilised in PBS. A ferricyanide (Fe^2+^/Fe^3+^) based redox probe was used for probing the electrochemical performance of the modified sensor. The rebinding of the template Tau-441 onto the electrode surface was performed by pipetting increasing concentrations of Tau-441 in PBS and ISF, respectively onto the electrodes and incubating for 30 min at room temperature. The calculation for the molarities is mentioned in the supplementary information (Table T1).

### 2.4 Sensing experiments

The preparation of different concentrations of Tau-441 were carried out by serial dilution of the stock solution using PBS, ISF, and SBF matrices. Initial ferricyanide probe-based measurements were carried out using the PANI_Tau-441_. The sensing experiments were carried out using differential pulse voltammetry (DPV) in a potential window of −0.2 V to +0.4 V. Initially, binding of Tau-441 to the PANI_Tau-441_ was carried out for a duration of 30 min. This was followed by performing DPV in a ferricyanide solution. These experiments were carried out in PBS and ISF matrices. For the probe free measurements, the SPGE surface was first modified with V_x_PDA following by the procedure for MIP formation. The measurements were carried out using DPV. Following the preparation of the V_x_PDA/PANI_Tau-441_ sensor surface different concentrations of Tau-441 were bound on the sensor surface. The binding was carried out for a duration of 30 min. The DPV was carried out using in the potential range −0.1 to +0.5 V at a scan rate of 0.05 V/s. The probe free experiments were first carried out in ISF followed by the use of SBF for the measurements using the handheld units.

The sensor response was plotted between signal loss and logarithm of concentration. The signal loss was calculated according to the formula:

(1) $$Signal\;Loss=\left(\frac{I_{i}-I_{0}}{I_{0}}\right)*100$$

where $I_{i}$ is the current response obtained from DPV for a particular concentration and $I_{0}$ is the current response obtained from DPV at baseline concentration.

The handheld unit was designed with point of care application in mind. The device has three main units, the microcontroller (μC), the analogue front end (AFE) and the display unit. The microcontroller is a ESP32 based board powered by a dual core LX6 system on a chip (SoC). A potentiostat AFE was chosen from Texas Instruments (LMP91000). The AFE and the SoC communicate through the I^2^C bus. The display unit consists of a 1.3″ 128 × 64 high contrast OLED display on the shared I^2^C bus. The SoC's internal 12-bit ADC is used to read the current values. The SoC was programmed to run voltammetry and communicate the data to an external machine running a python script through Universal Asynchronous Rx Tx (UART). An external python script is used to detect peaks in the voltammogram. The peak heights are then calculated and referenced with the calibration plot of the sensor to obtain the results. The result values are then communicated back to the device through UART. An FTDI bridge was used to bridge UART and USB Serial. An enclosure was 3D printed using an FDM 3D printer. The SPE connectors were obtained from RS components. Wired communication was used as a proof of concept for the working of the potentiostat.

### 2.5 Characterization

The electrochemical characterisation of the MIP and NIP were carried out using cyclic voltammograms and impedance spectra (EIS).

Morphological characterisation and elemental analysis of the vanadium MXenes (VMx) and VMx polydopamine (V_x_PDA) composites were carried out by using a JEOL JSM 7800F Field Emission scanning electron microscope (SEM) and an Oxford Instruments X-Max EDS detector.

Surface chemical composition of both the VMx and V_x_PDA was obtained using X-Ray Photoelectron Spectroscopy (XPS). A Kratos Axis Supra instrument using a monochromated Al Kα source with a 15 mA emission current and 225 W total power. In all cases wide scans were undertaken with a pass energy of 160 eV followed by high resolution data on the C 1s, O 1s, N 1s, and V 2p peaks with a pass energy of 40 eV, 0.1 eV step size and a dwell time of at least 2000 ms. Each sample was measured 5 times with a representative example displayed in this paper.

Quantification was carried out using CasaXPS (Version 2.3.24PR1.0) (Fairley et al., 2021), using Shirley backgrounds, with all scans being normalised against the C–C peak (284.8 eV). For C 1s, O 1s, and N 1s peaks a GL (30) line shape was used, with the V 2p peaks requiring a LA (1.2,5,8) due to their asymmetry (Biesinger et al., 2010).

## 3 Results and discussion

Fig. 1A shows the schematic of the formation of the MIP and its subsequent applications for sensing applications. Fig. S1A shows the CVs of the deposition of PANI MIP. Following the electropolymerisation process and template removal, Tau-441 is rebound onto the developed MIP for sensing applications using ferricyanide as the external redox probe. To compare the performance of the developed MIP, an NIP was developed. The NIP was composed of electropolymerized aniline only. Cyclic voltammetry (CV) was utilized to analyse the performance of these surfaces in using a ferricyanide redox. The MIP () and NIP () before removal of the template, exhibited current responses of 3.6276 μA and 5.98 μA, respectively. After treatment of both surfaces with oxalic acid, MIP () and NIP () surfaces exhibited approximately 66 μA and 55 μA, respectively (Fig. 1B). This increase in current response in the case of MIP could be attributed to the removal of the template, resulting in an increased surface area in contact with the ferricyanide solution (Ben Hassine et al., 2021; Stephen et al., 2023). However, in the case of NIP, this could be attributed to the interaction with oxalic acid, which results in the formation of conducting emeraldine when oxalic acid is drop casted on NIP as part of the template removal step (Erdem et al., 1996). This trend could also be observed in the impedance spectra that were obtained for these modifications (Fig. 1C and D (magnified image post template removal)). The EIS data shows both real and fit data.

**Fig. 1:**
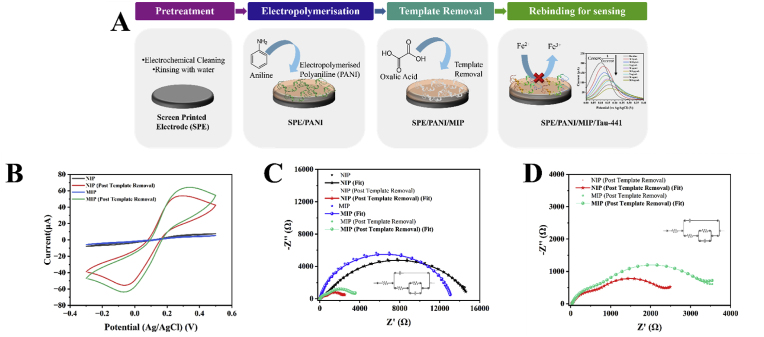
(A) Schematic of the application of PANI-Tau 441 MIP for the detection of Tau-441 using ferricyanide as the redox probe, (B) CV, (C) and (D) EIS spectra of the MIP and NIP modified SPEs in ferricyanide solution before and after template removal. (D) shows the magnified spectra for the MIP and NIP post template removal.

To assess the performance of the MIP and NIP surfaces in the presence of Tau-441 in PBS (pH 7.4), EIS studies were performed. These studies were performed for different concentrations of Tau-441. It could be observed that the NIP modified electrodes () were not able to detect variations in concentrations in a linear manner from 0 ng/mL (0 nM/L) to 500 ng/mL (12.2 nM/L), which implied that these electrodes could not be used for the detection of Tau-441 (Fig. S1 B). In contrast the MIP modified electrodes () were able to detect varying concentration from 0 ng/mL (0 nM/L) to 5000 ng/mL (122 nM/L) in a linear manner, which implied its application for the detection of Tau-441 in various matrices (Fig. S1 C). The EIS data included both fit and real data.

To analyse the presence of Tau-441 in various matrices, DPV was carried out in the potential range from −0.2 V to 0.4 V. Voltammetry was selected due to ease in fabricating commercial devices for the detection of protein biomarkers. It could be observed that the MIP based electrodes were able to detect Tau-441 concentrations from 50 fg/mL to 5 ng/mL (1.22 fM/L to 122 pM/L) in 1 mM PBS (Fig. 2A). The MIP based electrodes were then used or the detection of Tau-441 in ISF. These sensors were able to detect Tau-441 from 50 fg/mL to 500 ng/mL (1.22 fM/L to 12.2 nM/L) (Fig. 2C). The DPV based responses were attributed to the external redox mediator, which was used for the detection. The peaks in the DPV at approximately 0.1 V corresponding to the conversion of ferricyanide to ferrocyanide. Additionally, a slight shift could be observed in the DPVs both in PBS and also ISF. This could be attributed to the occupation of the MIP by the Tau-441, which resulted in the need for more potential for the conversion of the ferricyanide ions (El-Sharif et al., 2022; Lach et al., 2021). The calibration plots in Fig. 2B and D showed the normalised sensors response showing linear response for 6 orders of magnitude. For normalisation the base line response was recorded at approximately 210 μA.

**Fig. 2:**
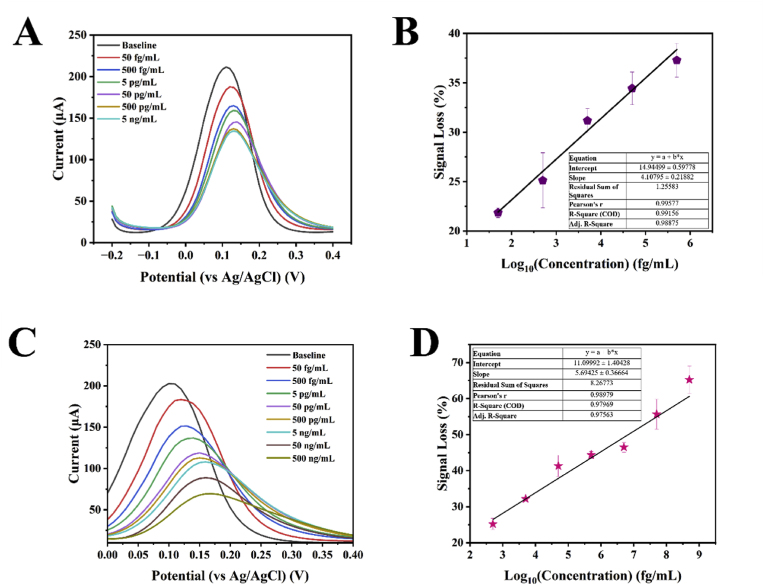
DPV of the MIP modified electrodes in different concentrations of Tau-441 in (A) 1 mM M PBS (pH 7.4) and (B) Artificial ISF (pH 7.4).

Although the detection of Tau-441 was carried out in ISF using ferricyanide as a redox mediator, this system presents some challenges based on the necessity of an external redox molecule for the detection of Tau-441. The use of a redox active layer beneath the MIP would help towards the commercialisation of devices, which could be used for the detection of biomarkers directly from ISF and serum. There have been extensive reports on the MIP based detection of proteins using ferricyanide based redox mediators (Ben Hassine et al., 2021; Choi et al., 2022; Eduarda Schneider et al., 2022). Additionally, proteins have also been detected using the sandwich hybridisation strategy (Li et al., 2020; Razzino et al., 2020). To the best of our knowledge, there has been only a single report on the detection of Tau-441 using a strategy involving an *in situ* redox probe. This report by Hassine et al. reported on the use of Prussian blue cubes as the redox probe for the detection of Tau-441 from PBS (pH 5.6). This sensor was able to detect Tau-441 in the range 1.09 pM/L to 2.18 pM/L with an LOD of 10 fM/L. In difference to existing reports, we have explored the use of polydopamine (PDA) as a redox probe for the detection of Tau-441. Additionally, this report also explored the use of VMx as a platform for the deposition of PDA. This composite was synthesized using a facile strategy, which involved the polymerisation of PDA in Tri-HCl in the presence of VMx.

To determine the morphology of the composite, SEM images of the pristine VMx were compared with the V_x_PDA composite. It could be observed from Fig. 3 A and B that the pristine MXene possesses an accordion like structure, which showed layers stacked above each other as reported by recent studies (Chen et al., 2023; Wu et al., 2024). This accordion-like structure also revealed separation of subsequent layers. On modification of the VMx with PDA, it could be observed from the SEM images (Fig. 3C) that the accordion like MXene were covered by a layer of PDA. From Fig. 3D, which is a magnified image of Fig. 3C, particles of PDA, which possess sizes of approximately 100 nm could be observed. EDS spectra of the V_x_PDA confirmed the presence of the V, C, F, O, and N in the composite (Fig. S2 A and B).

**Fig. 3:**
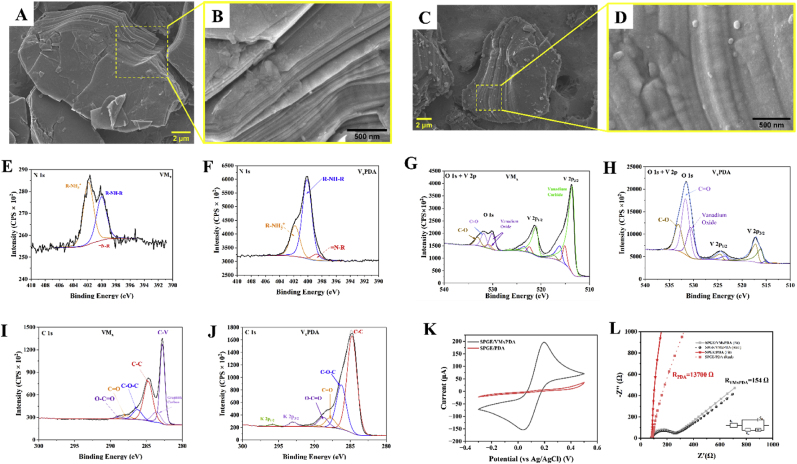
SEM image of (A) VMx showing accordion like sheets, (B) Magnified image of the sheet, (C) V_x_PDA composite, and (D) magnified image of the composite showing PDA covered VMx sheets. (E)–(J) XPS spectra of the VMx and V_x_PDA composites in the N1s, O 1s + V 2p, and C 1s regions. (K) CVs and (L) EIS of SPGEs modified with V_x_PDA () and PDA () showing decreased resistance of the V_x_PDA in ferricyanide solution. The EIS includes both fit and real data.

XPS signals of N (VMx and V_x_PDA; Fig. 3E and F, respectively), O and V (VMx and V_x_PDA; Fig. 3G and H, respectively), and C (VMx and V_x_PDA; Fig. 3I and J, respectively) were measured for the V_x_PDA film against an uncoated sample (VMx) and the large increase in N signal and changes in the C and O indicate successful coating of the PDA films on the VMx. The N 1s spectra of the V_x_PDA covered sheets were fitted with 3 primary peaks at 401.8, 400.1 and 398.8 eV, which have been assigned to R–NH_3_^+^, R–NH–R and = N–R, respectively (Ding et al., 2014; Hemmatpour et al., 2023).

The C 1s regions for the VMx and the V_x_PDA were modelled with the adventitious carbon as 4 components for the C–C, C–O–C (or C–N), O–C=O, and C=O, with positions at 284.8, 286.2, 288.7 and 287.8 eV, respectively (Biesinger, 2022). Additional peaks for vanadium carbide were modelled within the carbon, which were evidenced at 282.8 eV. These are most dominant in the uncoated samples as expected, with a matching shift in the V 2p peaks for a similar loss of detectable carbide (Ruiz et al., 2022).

The O 1s of the coated and uncoated material shows lower oxygen content in the uncoated samples. The PDA coated sample possess dominant peaks of C–O and C=O functional groups, which suggests both catechol (C–O, 533.2 eV) and Quinone (C=O, 531.8 eV) are present in the PDA structure (Wei et al., 2010). A reduction in the V 2p signal also shows evidence of the PDA coating being successfully applied, as well as the Vanadium Carbide to Vanadium Oxide shift being evident.

To verify the electrochemical performance of the synthesized material, CVs (Fig. 3K) and EIS (Fig. 3L) of the V_x_PDA and PDA were carried out ferricyanide solution. The CVs of the material showed an anodic peak of 200 μA, which could be attributed to the improved surface area of the composite, while the PDA film exhibited an anodic peak of only 10 μA indicating that the use of the MXene improved the loading of PDA, which led to current amplification. This could also be observed from the impedance spectra where in the PDA showed a resistance of 1.37 kΩ (Fig. 3J, ) while the V_x_PDA composite possessed a resistance of 154.8 Ω (Fig. 3J, ). The EIS includes both the fit and real values to show the agreement between these values.

**Fig. 4:**
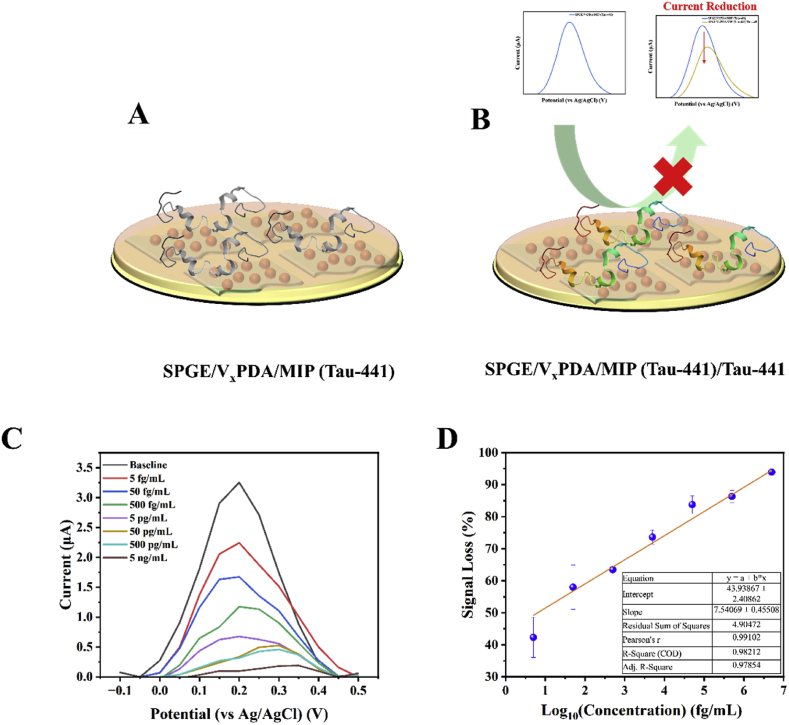
(A) Schematic of the detection of Tau-441 using SPGE modified with V_x_PDA and electropolymerized with PANI-Tau-441 MIP and (B) Reduced current due to the binding of Tau-441 as a result of binding of Tau-441. (C) CV and (D) EIS of the V_x_PDA modified SPGEs.

The V_x_PDA composite was drop casted on a SPGE followed by the deposition of the MIP. Consequently, the elution of the template by OA was carried out and the modified surface was then utilized for the detection of Tau-441 in ISF as shown in the schematic diagram in Fig. 4A. In Fig. 4B, the binding of the Tau-441 to the MIP led to a decrease in anodic peak current corresponding to the redox of the V_x_PDA.

The modified sensor was able to detect Tau-441 concentrations from 5 fg/mL to 5 ng/mL (122 aM/L to 122 pM/L) in ISF (Fig. 4C). The LOD exhibited by this sensor was 2.3 fg/mL (60 aM/L) (3.3 σ/S). The specific cavities possessed by MIPs aid in the capture of only analytes specific to these cavities (Li et al., 2024). Moreover, the phosphorylation of Tau proteins in AD, leads to alteration of conformation of the protein. This alteration in conformation lends support to making MIPs based sensors specific to only the target protein, which was used as the template (Nestler and Greengard, 1999). The analytical performance of this sensor was normalised as signal loss vs log of concentration. It could be observed that the sensor was able to detect Tau-441 concentrations over 6 orders of magnitude using the V_x_PDA composite (Fig. 4D). Additionally, the ISF used in this study contains common matrix interferents like glucose, NaCl and KCl, which shows the interference resistance capabilities of this sensor. Previous reports by Ben Hassine et al. have also shown that MIP based sensors are resistant to common interferents such as albumin and uric acid, which shows the interference resistance of MIP based sensors (Ben Hassine et al., 2021, 2023).

This method in contrast to existing reports utilizes only the sample matrix and functions without the need for an external redox probe. Additionally, the sensor was able to detect Tau-441 to the lowest concentration in comparison to existing reports as compared to reports in supplementary information Table T2 (Ben Hassine et al., 2021, 2023; Ren et al., 2023; Li et al., 2020; Nur Sonuç Karaboğa and Kemal Sezgintürk, 2023; Ye et al., 2020; Nangare and Patil, 2023; Toyos-Rodríguez et al., 2023; Sonuç Karaboga and Sezgintürk, 2020).

**Fig. 5:**
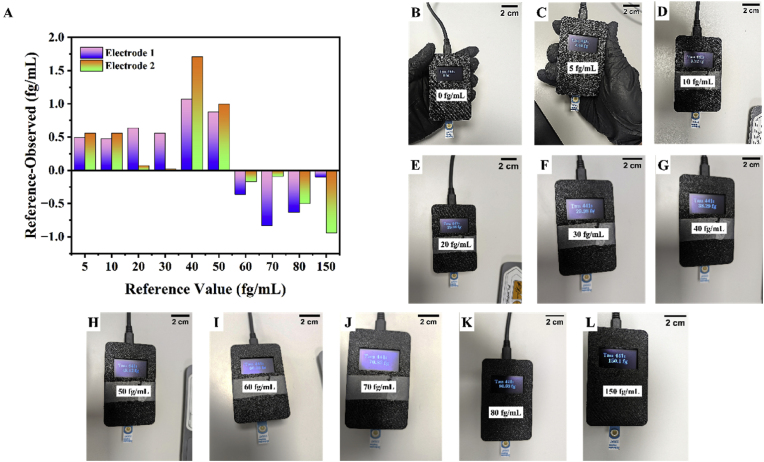
(A) Differences between reference value and observed value for the fabricated handheld device, (B–L) Photographs of the handheld device showing concentrations of the samples drop casted onto the electrode inserted into the device.

To verify the applicability of the sensor for the detection of Tau-441 from blood, we spiked various concentrations of Tau-441 in artificial serum. Additionally, as a step towards preparing commercialised point of care devices, we fabricated a handheld device, which was programmed to run voltammetry and detect peaks in the voltammogram. Photographs of the interior of the handheld device showing the circuit employed for its realisation is shown in Fig. S3 A and B. The circuit diagram showing the connections in this circuit is shown in Fig. S3 C. The peak heights are then calculated and referenced with the calibration plot of the sensor, which was obtained as part of Fig. 4B to obtain the results. The total materials cost involved in fabricating the device was found to be ∼ £20/device (supplementary information Table T3). Fig. 5A shows the differences between the values calculated by the device and the reference value that was dropped on the electrodes connected to the device. It can be observed that the differences between the reference value and the observed values were not significantly different and that the developed device could be used to predict the concentration of Tau-441 in artificial serum from 5 fg/mL to 150 fg/mL within a short time span of 32 min (30 min incubation and 2 min time for detection). Photographs of the results displayed by the device are shown in Fig. 5 B to L. A video showing the working of the device is attached with this report.

Future work in this direction will include the fabrication of devices, which are able to detect Tau protein concentrations in human blood and ISF. A web server for accessing, viewing, receiving, processing, and returning data will be fabricated as part of future reports. These devices will also include Bluetooth and WIFI connectivity.

## 4 Conclusion

Our study has successfully developed and characterized a novel sensor based on a redox active vanadium MXene polydopamine (V_x_PDA) composite for the ultrasensitive detection of Tau-441 in both interstitial fluid (ISF) and artificial serum. This sensor, utilizing a polyaniline-based molecularly imprinted polymer (PANI MIP), demonstrated a remarkably low detection limit of 2.3 fg/mL (∼60 aM/L) in ISF, confirming its potential for early-stage Alzheimer's disease diagnostics. The dynamic range of detection spans from 5 fg/mL to 5 ng/mL in ISF (122 aM/L to 122 pM/L) and 5 fg/mL to 150 fg/mL (122 aM/L to 366 aM/L) in serum, showcasing the sensor's robust applicability across different biological matrices. Additionally, the device possessed a low cost of ∼ £20 and could detect Tau-441 within a short span of 32 min, which is a good improvement in detection cost and time in comparison with gold standards. Importantly, this research underscores the feasibility of detecting neurodegenerative biomarkers in less invasive bodily fluids, aligning with the growing trend towards patient-friendly diagnostic methodologies. The handheld device we fabricated extends the practical utility of this sensor, making it a promising tool for point-of-care testing of Tau proteins, which are an important prognostic biomarker for AD.

Future studies will focus on validating this sensor in a clinical setting, exploring its responsiveness to Tau proteins in a broader population, and comparing its performance with existing diagnostic standards. Additionally, we also aim to develop a web server for accessing, viewing, receiving, processing, and returning data. Integrating this sensor technology into wireless wearable devices could further revolutionize the approach to monitoring and managing Alzheimer's disease, potentially allowing for real-time, continuous assessment of biomarker levels. The V_x_PDA/PANI MIP sensor represents a significant advancement in the biochemical detection of Alzheimer's biomarkers, with the potential to significantly impact early diagnosis and management of this debilitating condition.

## CRediT authorship contribution statement

**Ajith Mohan Arjun:** Writing – review & editing, Writing – original draft, Visualization, Validation, Methodology, Investigation. **Sudhaunsh Deshpande:** Validation, Software, Methodology, Investigation, Formal analysis, Data curation. **Tom Dunlop:** Writing – original draft, Investigation, Data curation. **Beth Norman:** Writing – original draft, Validation, Investigation. **Daniela Oliviera:** Writing – review & editing, Methodology, Investigation. **Georgeta Vulpe:** Writing – original draft, Validation, Investigation. **Felismina Moreira:** Writing – original draft, Validation, Resources, Methodology, Conceptualization. **Sanjiv Sharma:** Writing – review & editing, Writing – original draft, Visualization, Validation, Supervision, Project administration, Methodology, Investigation, Funding acquisition, Conceptualization.

## Declaration of competing interest

The authors declare that they have no known competing financial interests or personal relationships that could have appeared to influence the work reported in this paper.

## Figures and Tables

**figs1:**
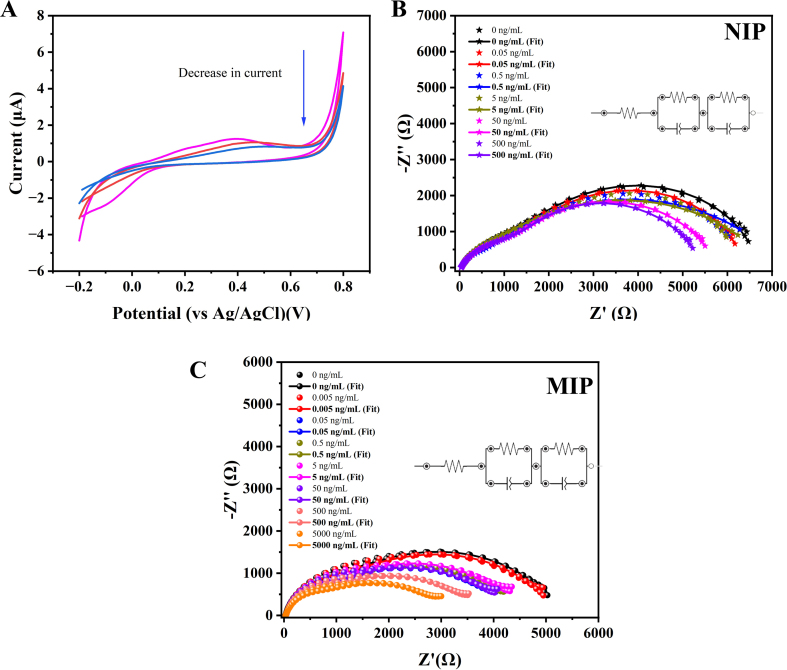


**figs2:**
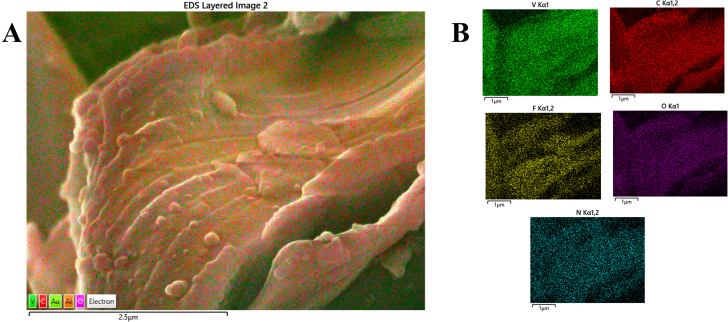


**figs3:**
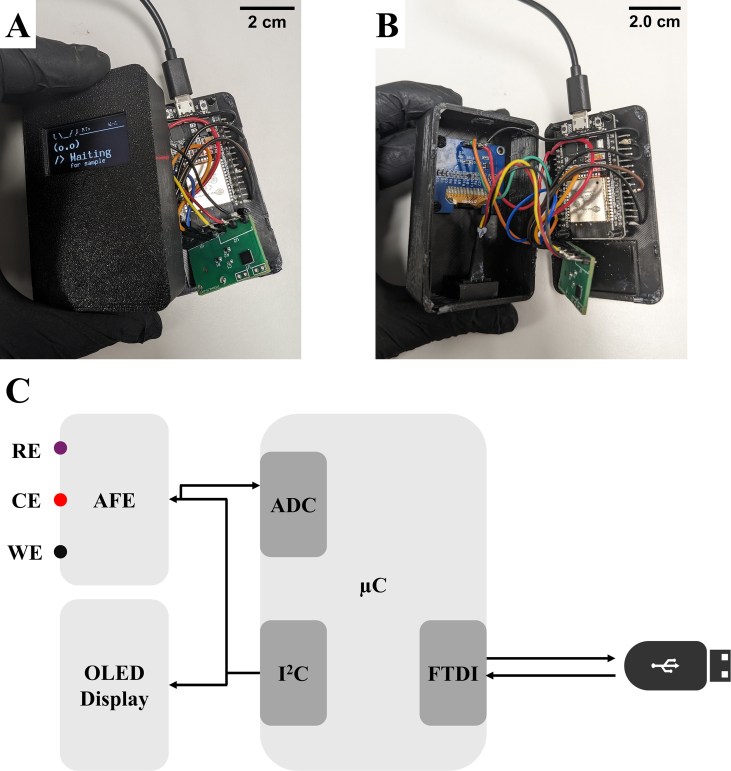


## Data Availability

Data will be made available on request.
