# Supplementary material for: Alzheimer's diagnosis beyond cerebrospinal fluid: Probe-Free Detection of Tau Proteins using MXene based redox systems and molecularly imprinted polymers
Source: Biosens Bioelectron X. 2024 Oct;20:100513. doi: 10.1016/j.biosx.2024.100513 (PMC11406148; doi:10.1016/j.biosx.2024.100513)
Supplement: Multimedia component 1 [file mmc1.docx]

**Alzheimer's diagnosis beyond cerebrospinal fluid: Probe-Free Detection of Tau Proteins using MXene based redox systems and molecularly imprinted polymers**

Ajith Mohan Arjun*^1^, Sudhaunsh Deshpande^1#^, Beth Norman ^1#^, Tom Dunlop^2^, Felismina Moreira^3^, Georgeta Vulpe^1^, Sanjiv Sharma*^1^

^1^ Dept. of Biomedical Engineering, Faculty of Science and Engineering, Swansea University, UK

^#^ Contributed equally

^2^ The Advanced Imaging of Materials (AIM) Facility, Faculty of Science and Engineering, Swansea University, UK

^3^ CIETI - LabRISE-School of Engineering, Polytechnic of Porto, R. Dr. António Bernardino de Almeida, 431, 4249-015 Porto, Portugal

* arjun.ajithmohan@swansea.ac.uk; sanjiv.sharma@swansea.ac.uk

| **Table T1**: Calculations of Molarity | |
| --- | --- |
| **Molecular weight of 441 is 41 kDa** | |
| 1 kDa = 1000 g/mol | |
| 41 kDa = 4329.86 g/mol | |
| **Link to calculator**: https://www.bioline.com/media/calculator/01_04.html | |
| **Weight/Volume** | **Molarity** |
| 1 µg/mL | 24.4 nM/L |
| 5 µg/mL | 122 nM/L |
| 500 ng/mL | 12.2 nM/L |
| 50 ng/mL | 1.2 nM/L |
| 5 ng/mL | 122 pM/L |
| 500 pg/mL | 12.2 pM/L |
| 50 pg/mL | 1.22 pM/L |
| 5 pg/mL | 122 fM/L |
| 500 fg/mL | 12.2 fM/L |
| 50 fg/mL | 1.22 fM/L |
| 5 fg/mL | 122 aM/L |

**Figure S1** (A) CVs showing the deposition of the PANI MIP. (B) Impedance spectra showing the comparison of the detection of Tau-441 for NIP (▬) and MIP (▬) in 1 mM PBS (pH 7.4)


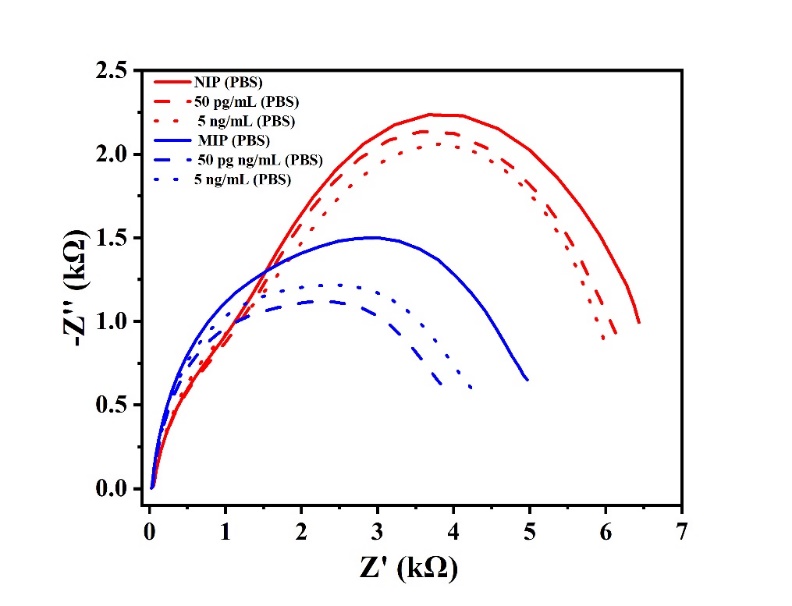

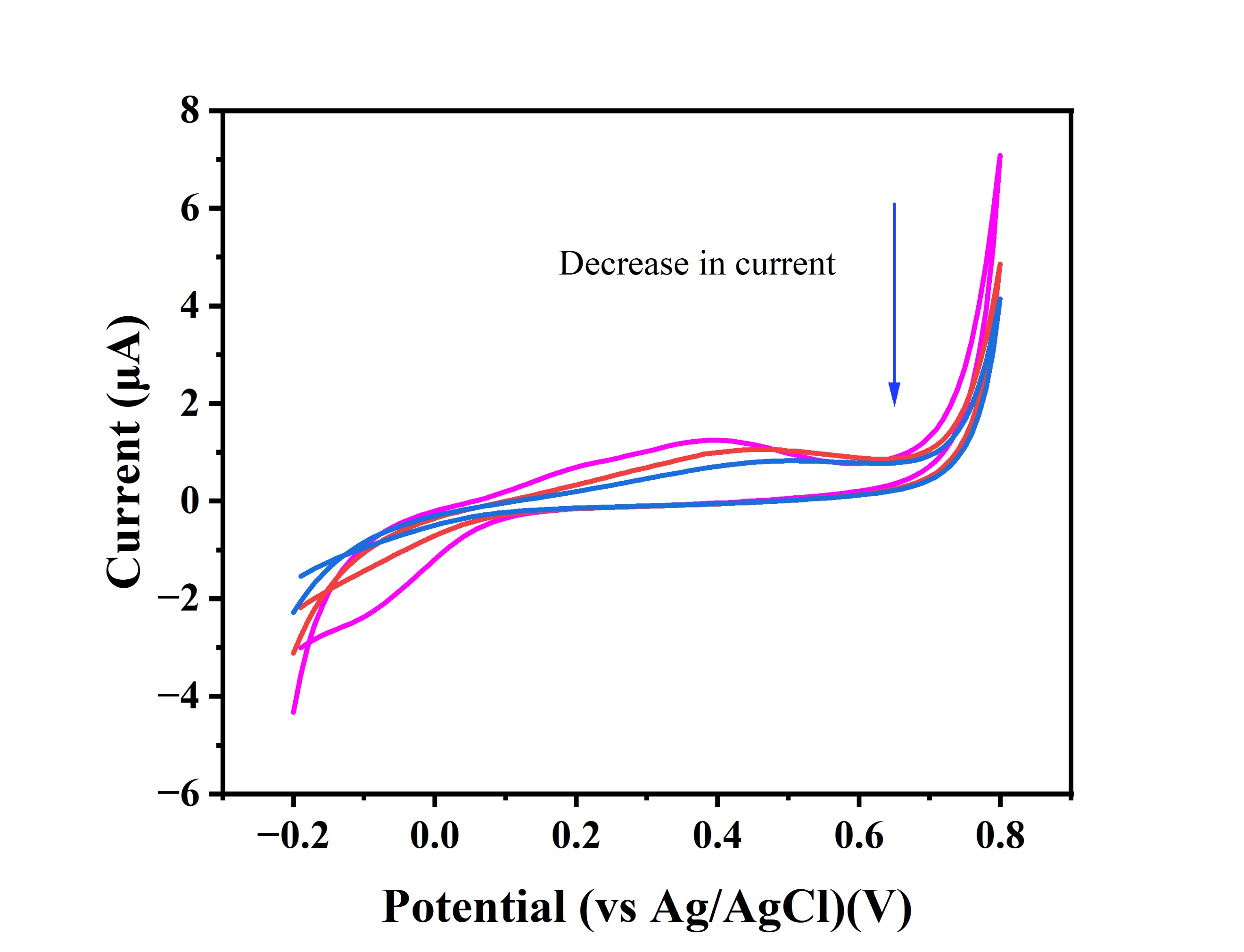


**A**

**B**


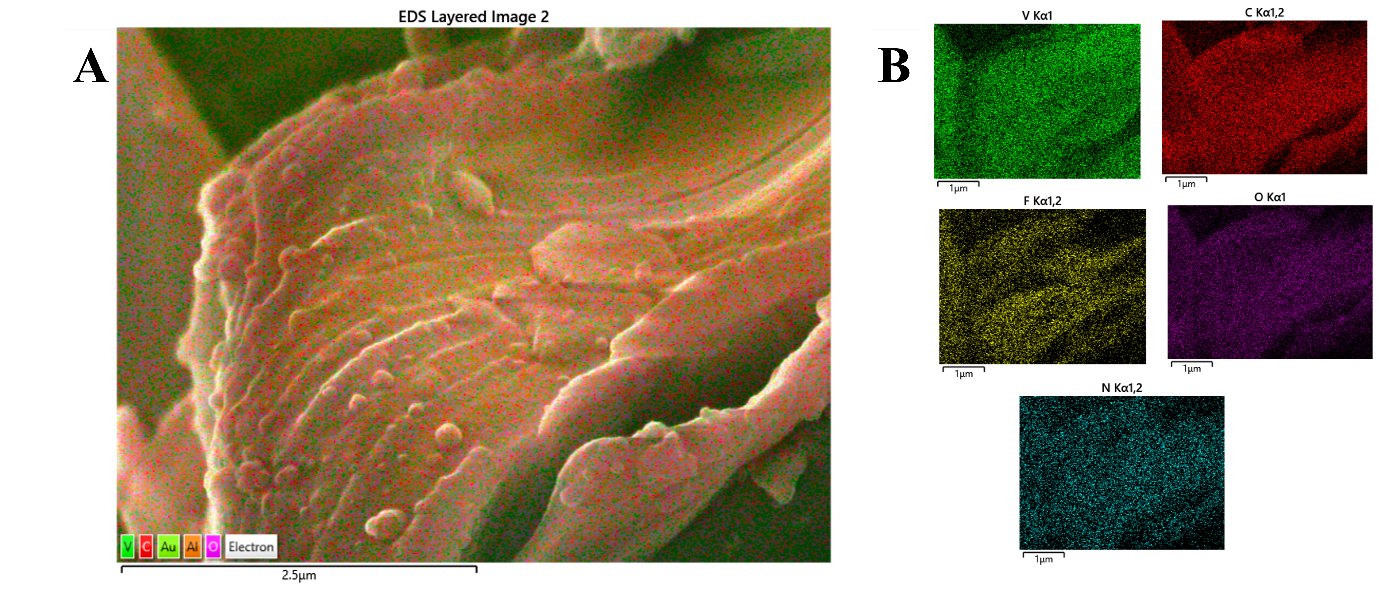


**Figure S2** (A) SEM micrograph of the area for EDS mapping. (B) Mapping of V, C, F, O, and N for the selected area for the V_x_PDA composite


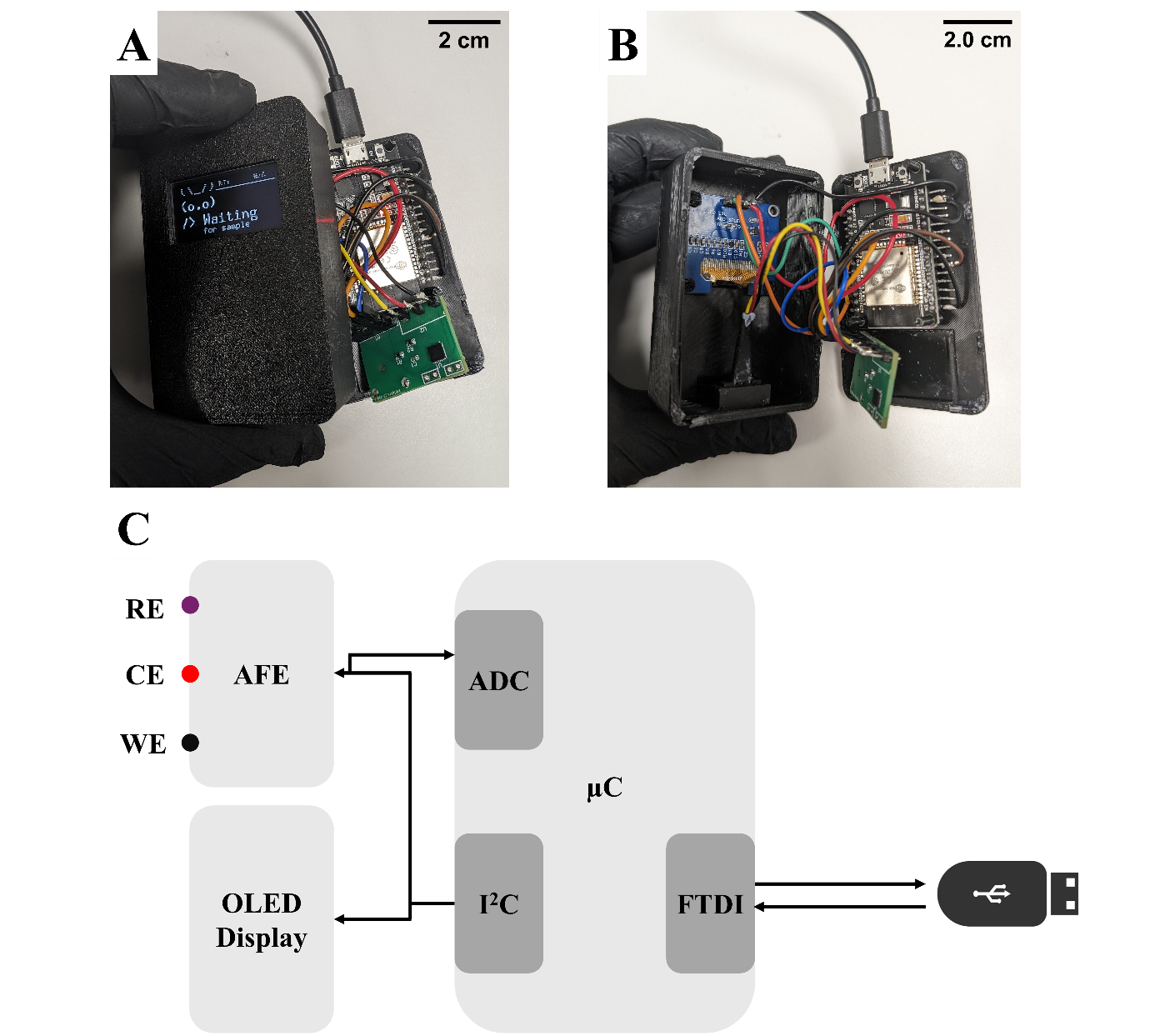


**Figure S3** (A) and (B) Photographs of the interior of the devices. **(C)** Circuit diagram showing the connections in this circuit.

| **Table T2:** Comparison of the developed sensor with other reports in literature | | | | | | | |
| --- | --- | --- | --- | --- | --- | --- | --- |
| **No** | **Analyte/Matrix** | **Material** | **Probe** | **Range** | **LOD** | **Ref** | **Remarks** |
| 1 | Tau-441 in Buffer | 3-aminophenol (3-AMP) (MIP) (C-SPCE/PBNC/GO/MIP) | Prussian Blue (PBNC) | 1.09-2.18 nM/L | 0.01 pM/L | (Ben Hassine et al. 2023) | **Probe Free Measurement** |
| 2 | Tau-441 in Buffer/Serum | 3-AMP (MIP) (C-SPE) | Ferricyanide (External) | 2.18 pM/L-2.18 nM/L | 0.02 pM/L | (Ben Hassine et al. 2021) |  |
| 3 | Au modified Tau-441 in Buffer/Serum of people with dementia | MWCNT-rGO-CS (Au NP conjugated to Tau-441) | Ferricyanide (External) | 0.5 fM/L-80 fM/L | 0.46 fM/L | (Li et al. 2020) | **Lowest Tau-441** |
| 4 | Tau-441 in Buffer/Serum | PEDOT: PSS/MWCNTs-COOH/Tau-441 antibody | Ferricyanide (External) | ~244 fM/L-1.2 µM/L (10 pg/mL-50 µg/mL) | ~122 fM/L (7.4 pg/mL) | (Ren et al. 2023) |  |
| 5 | Tau-441 in Buffer/Serum | SPCE/APTES/Tau-441 antibody | Ferricyanide (External) | ~122 fM/L- ~20 pM/L (6.4 pg/mL-800 pg/mL) | ~122 fM/L (5.3 pg/mL) | (Nur Sonuç Karaboğa and Kemal Sezgintürk 2023) |  |
| 6 | Tau-441 in Buffer/Serum | GCE/rGO/Tau-441 Antibody | Ferricyanide (External) | 80 fM/L-80 pM/L | ~75 fM/L | (Ye et al. 2020) |  |
| 7 | Tau-441 Spiked/Animal Sample | AuNP/LbL/Tau-441 Antibody | Optical Sensor | ~3.66 nM/L-0.122 fM/L (150 ng/mL-5 fg/mL) | ~7 fM/L (13.25 fg/mL) | (Nangare and Patil 2023) |  |
| 8 | Buffer/Serum samples | ITO/PET/Tau-441 Antibody | Ferricyanide (External) | ~0.122 nM/L-2.44 nM/L (5-100 ng/mL) | ~ 122 pM/L (4.3 ng/mL) | (Toyos-Rodríguez et al. 2023) |  |
| 9 | Buffer/Serum/CSF | ITO/Au/MUA (Thiol)/Tau-441 Antibody | Ferricyanide (External) | ~24.4 fM/L-12.2 pM/L (1- 500 pg/mL) | ~1.22 fM/L (91 fg/mL) | (Sonuç Karaboga and Sezgintürk 2020) |  |
| 10 | Blood serum | SPE/HBN/PDA/Tau antibody | Ferricyanide (External) | ~ 24.4 fM/L-732 fM/L (1-30 pg/mL) | ~12.2 fM/L (0.42 pg/mL) | (Er Zeybekler 2023) |  |
| **11** | **ISF and Serum** | **SPGE/VxPDA/PANI(Tau-441)** | **Polydopamine (PDA) (Internal)** | **122 aM/L-122 pM/L (5 fg/mL to 5 ng/mL)** | **~ 60 aM/L (2.3 fg/L)** |  | **This Work** |

| **Table T3:** Cost of device (Components wise) | | | |
| --- | --- | --- | --- |
| **No** | **Item** | **Source** | **Cost** |
| 1 | ESP32 based board powered by a dual core LX6 | <https://www.amazon.co.uk/ESP-32S-Development-2-4GHz-Bluetooth-Antenna/dp/B071JR9WS9> | £7 |
| 2 | Connecting Wires | <https://www.digikey.co.uk/en/products/detail/digilent,-inc./240-119/9445915?utm_adgroup=&utm_source=google&utm_medium=cpc&utm_campaign=PMAX%20Shopping_Product_Low%20Performers&utm_term=&productid=9445915&utm_content=&utm_id=go_cmp-19789935775_adg-_ad-__dev-c_ext-_prd-9445915_sig-Cj0KCQjw6PGxBhCVARIsAIumnWaDgVpQXNFabycOdPIwwCJhSy8UniFwbPvPri3W38nvCOgk9X9G5l4aAj67EALw_wcB&gad_source=1&gclid=Cj0KCQjw6PGxBhCVARIsAIumnWaDgVpQXNFabycOdPIwwCJhSy8UniFwbPvPri3W38nvCOgk9X9G5l4aAj67EALw_wcB> | £4 |
| 3 | Potentiostat AFE (LMP91000) | [https://www.ti.com/product/LMP91000#order-quality](https://www.ti.com/product/LMP91000%23order-quality) | £3 |
| 4 | 1.3” 128×64 high contrast OLED display | <https://www.amazon.co.uk/128X64-Serial-Display-Module-Color/dp/B075H3YGBZ> | £7 |
| **Total** | | | **£21** |

Ben Hassine, A., Raouafi, N., Moreira, F.T.C., 2021. Novel Electrochemical Molecularly Imprinted Polymer-Based Biosensor for Tau Protein Detection. Chemosensors.

Ben Hassine, A., Raouafi, N., Moreira, F.T.C., 2023. Novel biomimetic Prussian blue nanocubes-based biosensor for Tau-441 protein detection. Journal of Pharmaceutical and Biomedical Analysis 226, 115251.

Er Zeybekler, S., 2023. Polydopamine-coated hexagonal boron nitride-based electrochemical immunosensing of T-Tau as a marker of Alzheimer's disease. Bioelectrochemistry 154, 108552.

Li, X., Jiang, M., Cheng, J., Ye, M., Zhang, W., Jaffrezic-Renault, N., Guo, Z., 2020. Signal multi-amplified electrochemical biosensor for voltammetric determination of tau-441 protein in biological samples using carbon nanomaterials and gold nanoparticles to hint dementia. Microchimica Acta 187(5), 302.

Nangare, S., Patil, P., 2023. Poly(allylamine) coated layer-by-layer assembly decorated 2D carbon backbone for highly sensitive and selective detection of Tau-441 using surface plasmon resonance biosensor. Anal. Chim. Acta 1271, 341474.

Nur Sonuç Karaboğa, M., Kemal Sezgintürk, M., 2023. A Practical Approach for the Detection of Protein Tau with a Portable Potentiostat. Electroanalysis 35(3), e202200072.

Ren, H., Liu, X., Wei, S., Zhao, F., Chen, Z., Xiao, H., 2023. An Electrochemical Immunosensor with PEDOT: PSS/MWCNTs-COOH Nanocomposites as a Modified Working Electrode Material for Detecting Tau-441. Chemosensors.

Sonuç Karaboga, M.N., Sezgintürk, M.K., 2020. Analysis of Tau-441 protein in clinical samples using rGO/AuNP nanocomposite-supported disposable impedimetric neuro-biosensing platform: Towards Alzheimer's disease detection. Talanta 219, 121257.

Toyos-Rodríguez, C., García-Alonso, F.J., de la Escosura-Muñiz, A., 2023. Towards the maximization of nanochannels blockage through antibody-antigen charge control: Application for the detection of an Alzheimer’s disease biomarker. Sensors Actuators B: Chem. 380, 133394.

Ye, M., Jiang, M., Cheng, J., Li, X., Liu, Z., Zhang, W., Mugo, S.M., Jaffrezic-Renault, N., Guo, Z., 2020. Single-layer exfoliated reduced graphene oxide-antibody Tau sensor for detection in human serum. Sensors Actuators B: Chem. 308, 127692.

**References**
